# Hippocampal-Dependent Antidepressant Action of the H_3_ Receptor Antagonist Clobenpropit in a Rat Model of Depression

**DOI:** 10.1093/ijnp/pyv032

**Published:** 2015-04-27

**Authors:** Teresa Femenía, Salvatore Magara, Caitlin M. DuPont, Maria Lindskog

**Affiliations:** Department of Neuroscience, Karolinska Institutet, Stockholm, Sweden (Drs Femenía, Magara, and Lindskog, and Ms DuPont).

**Keywords:** Anxiety, FSL, glutamate, memory, plasticity

## Abstract

**Background:**

Histamine is a modulatory neurotransmitter regulating neuronal activity. Antidepressant drugs target modulatory neurotransmitters, thus ultimately regulating glutamatergic transmission and plasticity. Histamine H_3_ receptor (H_3_R) antagonists have both pro-cognitive and antidepressant effects; however, the mechanism by which they modulate glutamate transmission is not clear. We measured the effects of the H_3_R antagonist clobenpropit in the Flinders Sensitive Line (FSL), a rat model of depression with impaired memory and altered glutamatergic transmission.

**Methods:**

Behavioral tests included the forced swim test, memory tasks (passive avoidance, novel object recognition tests), and anxiety-related paradigms (novelty suppressed feeding, social interaction, light/dark box tests). Hippocampal protein levels were detected by Western blot. Hippocampal plasticity was studied by in slice field recording of CA3-CA1 long-term synaptic potentiation (LTP), and glutamatergic transmission by whole-cell patch clamp recording of excitatory postsynaptic currents (EPSCs) in CA1 pyramidal neurons.

**Results:**

Clobenpropit, administered systemically or directly into the hippocampus, decreased immobility during the forced swim test; systemic injections reversed memory deficits and increased hippocampal GluN2A protein levels. FSL rats displayed anxiety-related behaviors not affected by clobenpropit treatment. Clobenpropit enhanced hippocampal plasticity, but did not affect EPSCs. H_1_R and H_2_R antagonists prevented the clobenpropit-induced increase in LTP and, injected locally into the hippocampus, blocked clobenpropit’s effect in the forced swim test.

**Conclusions:**

Clobenpropit’s antidepressant effects and the enhanced synaptic plasticity require hippocampal H_1_R and H_2_R activation, suggesting that clobenpropit acts through disinhibition of histamine release. Clobenpropit reverses memory deficits and increases hippocampal GluN2A expression without modifying anxiety-related phenotypes or EPSCs in CA1 pyramidal neurons.

## Introduction

Depressive disorders are highly prevalent diseases that present both emotional and cognitive symptoms. About 50% of the patients with depression do not respond to treatment, and residual symptoms include cognitive deficits (McClintock et al., 2011). Because commonly used antidepressant drugs increase the levels of neuroamines, such as serotonin and norepinephrine, the neurobiology of depression was initially attributed to a deficit in the monoaminergic system (Hirschfeld, 2000). However, little evidence is available to suggest that altered levels of monoamines contribute to the etiology of depression. On the other hand, recent evidence suggests that depression may arise from a dysregulation of glutamatergic transmission and plasticity (Sanacora et al., 2012). Accordingly, the classic antidepressants that act on monoamines might exert their effect by modulating glutamatergic transmission and plasticity (Berton and Nestler, 2006).

Unlike the serotonergic and noradrenergic systems, histaminergic modulation in the central nervous system has not been studied thoroughly. Histaminergic neurons are located in the tuberomammillary nucleus in the hypothalamus, and these neurons project to many cerebral regions. Four subtypes of G-protein‒coupled histamine receptors (H_1_R, H_2_R, H_3_R, and H_4_R) are distributed widely throughout the brain (Haas and Panula, 2003; Connelly et al., 2009). H_3_ receptors are constitutively active presynaptic auto- and hetero-receptors (Schwartz, 2011), that inhibit release of histamine and have also been shown to reduce glutamate release (Brown and Haas, 1999; Molina-Hernandez et al., 2001; Garduno-Torres et al., 2007). H_3_R activation decreases gamma oscillations (Andersson et al., 2010), which have been correlated positively with cognition (Jensen et al., 2007). Consistent with the effect on gamma oscillations, an extensive battery of studies has revealed that H_3_R antagonists improve attention and memory (Passani et al., 2004; Esbenshade et al., 2008; Sander et al., 2008), whereas H_1_ and H_2_R antagonists inhibit memory formation (Gianlorenco et al., 2014; Taati et al., 2014). However, the role of histamine receptors in depression has been less explored and never confirmed in an animal model of depression. Pérez-García et al. (1999) reported a decrease in the behavioral despair in mice when administered with H_3_R antagonists thioperamide or clobenpropit, suggesting an antidepressant effect. In addition, a recent characterization of new non-imidazole histamine H_3_R antagonists has revealed antidepressant activities in the forced swim test in mice (Gao et al., 2013; Bahi et al., 2014). Because of their combined antidepressant and pro-cognitive benefits, H_3_R antagonists are promising candidates for treating both the emotional and cognitive symptoms of depression. However, the mechanism underlying the therapeutic action of H_3_R antagonism is poorly understood in the context of a depression model.

The complex symptomatology of depression reflects the involvement of several brain regions in the disorder’s pathophysiology. The importance of the hippocampus in depression is supported by the finding that patients with depression have reduced hippocampal volume (Campbell et al., 2004; Stockmeier et al., 2004). Moreover, the hippocampus is increasingly recognized as the brain’s integrator of emotions and cognition (Small et al., 2011).

The Flinders Sensitive Line (FSL) is an inbred rat model of depression (Overstreet and Wegener, 2013); moreover, these rats have impaired emotional and recognition memory (shown in the passive avoidance and the novel object recognition tests, respectively; Eriksson et al., 2011; Gómez-Galán et al., 2013). Recent work in our laboratory has shown that FSL rats have both increased glutamatergic transmission and decreased long-term potentiation (LTP) in the hippocampus (Gómez-Galán et al., 2013). Thus, FSL rats are a suitable animal model for studying H_3_ receptor modulation of glutamate transmission for the emotional and cognitive symptoms of depression. Here, we report that treating FSL rats with the H_3_R antagonist clobenpropit reduces immobility in the forced swim test and improves memory, but does not affect anxiety-related behaviors. Moreover, clobenpropit treatment increases synaptic plasticity in the hippocampus without affecting basal synaptic transmission.

## Methods

### Animals and Husbandry

All experiments were performed using male Sprague-Dawley rats (total n = 49; Charles-River Laboratories) or male FSL rats (total n = 164; bred in-house). The rats were 2–3 months of age at the time of behavioral testing and their body weight was between 320–420g for the Sprague-Dawley group and 280–400g for the FSL group. Patch-clamp experiments were performed using 17‒23-day-old rats. The animals were group-housed under standard laboratory conditions (20–22°C, 50–60% humidity); animals that underwent surgery were single-housed. For the behavioral testing, the rats were handled for a minimum of 6 days before testing to minimize stress effects. Each animal was used for one test only. All experiments were approved by the Stockholm North Committee on Ethics of Animal Experimentation.

### Drugs

Clobenpropit, trans-triprolidine, cimetidine, and histamine were purchased from Tocris Bioscience. For behavioral experiments, clobenpropit was dissolved in saline and administered subcutaneously 45 minutes before the experiment at 5mg/kg unless otherwise stated. For hippocampal delivery, the drugs were dissolved in artificial cerebrospinal fluid (ACSF) and injected via bilateral guide cannulae using a microsyringe pump (1 μl/hemisphere) 15 minutes before the experiment at the following final concentrations: clobenpropit, 10mM; trans-triprolidine, 0.5mM; and cimetidine, 1mM. Drug doses and concentrations were chosen based on previous studies (Pérez-García et al., 1999; Xu et al., 2009). For slice electrophysiology, the drugs were dissolved in ACSF at the following final concentrations: clobenpropit, 10 μM; trans-triprolidine, 2 μM; cimetidine, 50 μM; and histamine, 10 μM. Final concentrations were chosen based on pilot experiments and previous studies (Zhou et al., 2006; Chepkova et al., 2012).

### Forced Swim Test

Immobility in the forced swim test is used to measure behavioral despair and has good predictive value for testing antidepressant effects (Porsolt et al., 1977). Each rat (n = 5–7 rats/group) was placed for 10min in a vertical Plexiglas cylinder (height: 50cm; diameter: 30cm) containing 37cm of water (25±1°C). Twenty-four hours later, the rat was placed in the cylinder for a second session and filmed for 5min. Rats were treated before each forced swim test session. Immobility time was measured by an observer who was blinded with respect to the experimental conditions and is defined as the cessation of activity aside from the absolute minimum movement required to remain afloat.

### Novel Object Recognition

The novel object recognition test was performed in a Plexiglas box (length: 80cm; width: 35cm; height: 35cm) containing two objects constructed out of plastic toys with different visual contrast and shape. A control experiment (not shown) showed that the rats had no clear preference for any of the objects used. The rat was habituated to the test box for 20min the day before the training (session S0). Rats were treated before the training session (S1) and then left to freely explore two identical objects for 5min. The baseline locomotor activity (as total distance covered and velocity) was measured during the training session by EthoVision system (XT 10.0, Noldus Inc.). After 24 hrs the rats (n = 5 rats/group) were returned to the box for a 5min test session (S2), with one of the objects replaced by a novel object (Figure S1A). Each trial was filmed, and an observer who was blinded with respect to the experimental conditions measured object exploration time. Exploration was defined as sniffing, biting, licking, or touching the object with the nose while facing it. A recognition index was then calculated for S2 using the formula [N/(N+F)], where N is the total time spent exploring the novel object and F is the total time spent exploring the familiar object.

### Passive Avoidance

Passive avoidance was measured as previously described (Luttgen et al., 2005). In brief, we used a commercial set-up comprised of a box with a light and a dark compartment separated by an automatic sliding door (TSE Systems). Rats were treated before the training session and then contained in the light compartment for 2min (Figure S1B), after which the sliding door was opened. After the rat entered the dark compartment, the sliding door was closed, and a weak electrical stimulus was delivered through the grid floor. Twenty-four hours later, the rat (n = 5–13 rats/group) was again placed in the light compartment, and the latency to enter the dark compartment (with all four paws) was measured; the cut-off time for the test was 9min.

### Novelty Suppressed Feeding Test

The novelty suppressed feeding test was performed in an open field (length: 80cm; width: 35cm; height: 35cm) containing a single food pellet placed on a white paper in the center of the cage. After 16h of food deprivation, the rat (n = 4–7 rats/group) was placed in the corner of the testing apparatus and allowed to explore the field for 5min. The latency time until the rat began to eat and the amount of food consumed were measured.

### Social Interaction

In the social interaction test, two rats of a similar strain (n = 7 rats/group) and treatment (but from different home cages) were placed together in an open field. The total time that the two rats interacted socially (i.e. sniffing, following, grooming, kicking, crawling under or over each other, and touching or nearly touching their faces) was measured for 5min (Femenía et al., 2011).

### Light/Dark Box Test

The light/dark box test was performed using the passive avoidance box, with the grid floor replaced with a solid Plexiglas floor. The total time spent in the light compartment and the number of transitions between the light and dark compartments were measured during a single 5-min trial using an integrated infrared beam system (n = 7 rats/group; Femenía et al., 2011).

### Surgery

Rats were anesthetized with isoflurane, and bilateral guide cannulae (Plastics One) were implanted above the CA1 field of the dorsal hippocampus at the following coordinates relative to bregma and the dura surface: mediolateral: ±3.0mm; anteroposterior: −4.2mm; and dorsoventral: −1.3mm; at a 0° angle from the vertical axis in the coronal plane (Paxinos and Watson, 1998). The implantation sites were confirmed histologically post-mortem (Figure S1C).

### Western Blot Analysis

4 hrs after treatment the rats were decapitated, and the hippocampi were rapidly dissected and sonicated in buffer containing 1% sodium dodecyl sulphate (SDS) and protease inhibitors (Roche). Immunoblot analysis was performed using standard protocols and analyzed using the LICOR Odyssey fluorescence detection system with fluorescent secondary antibodies. The data were quantified using ImageJ software (NIH), and expression was normalized to ß-actin or vinculin. The following primary antibodies were used: GluA1, GluA2, ß-actin, vinculin (Millipore); GluNR1 (Synaptic Systems); GluNR2A (Tocris Bioscience); GluNR2B, glutamate/aspartate transporter GLAST (human excitatory amino acid transporter EAAT1), and glial glutamate transporter GLT1 (human EAAT2) (Abcam).

### Field Recordings

Horizontal hippocampal slices (400 μM) were prepared as previously described (Gómez-Galán et al., 2013). ACSF contained (in mM): 130 NaCl, 3.5 KCl, 1.25 NaH_2_PO_4_, 24 NaHCO_3_, 10 glucose, 2 CaCl_2_, and 1 MgCl_2_ (pH 7.3–7.4, 310–330 mOsm). For recording, hippocampal slices were placed in a submersion chamber perfused with oxygenated ACSF (perfusion rate 2–3ml/min). Field excitatory postsynaptic potentials (fEPSPs) were elicited in the CA1 stratum radiatum by stimulating the Schaffer collateral pathway. Responses were collected every 60 s using a stimulation intensity that yielded 50–60% of either the maximal response or the appearance of population spikes (intensity range: 7‒16 μA). LTP was induced by high-frequency stimulation (HFS; three 1-s trains, 20 s apart, at a frequency of 100 Hz). The signal was filtered at 5kHz and sampled at 10kHz using a Digidata 1440A D/A converter and Axoscope 10.2 (Molecular Devices). fEPSP slope was measured using AxoGraph X as the maximum slope from 10 regression points over a 1-msec period during the constant rising phase of the fEPSP. The values were normalized to the average fEPSP slope obtained during the 10-min baseline period prior to HFS.

### Whole-Cell Recordings

Horizontal hippocampal slices (350 μm) were prepared as previously described (Gómez-Galán et al., 2013). The dissection solution contained (in mM): 250 sucrose, 2.5 KCl, 1.4 NaH_2_PO_4_, 26 NaHCO_3_, 10 glucose, 1 CaCl_2_, and 4 MgCl_2_ (310–330 mOsm). The recovery and recording ACSF solution contained (in mM): 130 NaCl, 3.5 KCl, 1.25 NaH_2_PO_4_, 24 NaHCO_3_, 10 glucose, 2 CaCl_2_, and 1.3 MgCl_2_. Recordings were done in a submersion chamber at 32–34°C with a perfusion rate of 2–3ml/min. Glass pipettes with a tip resistance of 3–6 MOhm were filled with a solution containing (in mM): 110 K-gluconate, 10 KCl, 4 Mg-ATP, 10 Na_2_-phosphocreatine, 0.3 Na-GTP, 10 4-(2-hydroxyethyl)piperazine-1-ethanesulfonic acid (HEPES), and 0.2 ethylene glycol tetraacetic acid (EGTA) (pH 7.2–7.4; 270–290 mOsm). Recordings were performed using a MultiClamp 700B and Clampex 10.0 (Molecular Devices), with filter and sampling rates of 5 and 10kHz, respectively (Digidata 1440A). Pyramidal neurons located in the CA1 pyramidal cell layer were voltage-clamped at -65 mV, and pipette capacitance was compensated. Access resistance was monitored, and cells with either a change in resistance >30% or membrane potential more positive than -55 mV were excluded from the analysis. Excitatory postsynaptic currents (EPSCs) were analyzed off-line using Mini Analysis software (Synaptosoft Inc.), with a low-pass cut-off elliptic filter at 1000 Hz; the amplitude threshold was 8 pA, and the area threshold was 20 pA/ms. Events were excluded if the rise time exceeded the decay time.

### Data Analysis

The two-tailed Student’s *t*-test was used to compare two groups and one-way analysis of variance (ANOVA; followed by Dunnett’s Multiple Comparison Test post hoc analysis, where applicable) was used to compare clobenpropit treatments at different doses with the saline-treated FSL group. Two-way ANOVA (followed by Bonferroni’s post hoc analysis, where applicable) was used to test for the interaction between clobenpropit treatment and H_1_/H_2_ receptor blockade. The Fisher’s exact test or the Chi-square test for trend (for two or more independent variables, respectively) was used when within-group variability was absent due to latency times exceeding the time of observation (in the passive avoidance and in the novelty suppressed feeding tests). The dependent variables were the number of rats performing (or not) the tested behavior. The Kolmogorov-Smirnov test was used to compare the cumulative distributions of EPSC inter-event intervals and amplitudes.

Data were analyzed using GraphPad Prism 5.0 (GraphPad Software Inc.) or JMP11 software (SAS).

## Results

### The Antidepressant Effect of Clobenpropit Involves the Hippocampus

**Figure 1.**
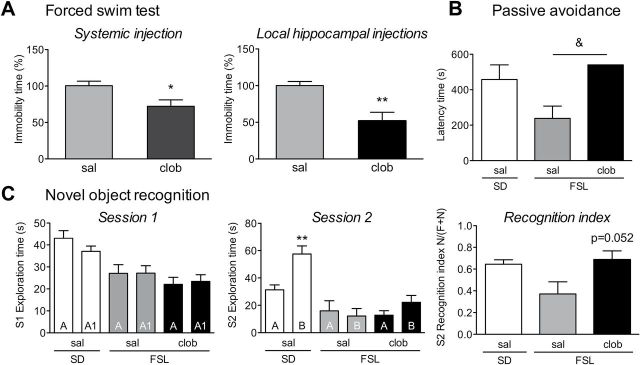
Clobenpropit reverses depressive behavior and cognitive deficits in Flinders Sensitive Line (FSL) rats. (A) Forced swim test. Total immobility time was measured during a 5-min trial and is expressed as the percentage of saline-treated FSL rats. Clobenpropit treatment (clob) decreased immobility time when given either systemically (left) or directly into the CA1 region of the hippocampus (right). *p < 0.05; **p < 0.01 vs. FSL saline (sal; Student’s *t*-test). (B) Passive avoidance test. Latency time (in seconds) to enter the compartment where the aversive stimulus had been given. Systemic clobenpropit treatment (5mg/kg, s.c.) increased the latency in FSL rats to Sprague-Dawley (SD) levels. ^&^ *p* < 0.05 (Fisher’s exact test). Note that none of the clobenpropit-treated FSL rats crossed to the aversive compartment within the 540-s observation period, explaining the lack of an error bar in this group. (C) Novel object recognition test. Total object exploration time (in seconds) was measured for 5min during session 1 (S1; left) and session 2 (S2; middle). In session 1, the two objects were identical and are referred to as “A” and “A1”; in session 2, object A1 was replaced with a novel object (“B”). In session 2, the saline-treated Sprague-Dawley rats spent significantly more time exploring the novel object than the familiar object (**p < 0.01, Student’s *t*-test). The right panel summarizes the recognition index measured during session 2, which was calculated as time spent exploring the novel object (N), divided by total exploration time of the familiar (F) and the novel (N) objects [N/(N+F)]. Treating FSL rats with clobenpropit restored the recognition index to Sprague-Dawley levels (*p* = 0.052 vs. FSL sal, Student’s t-test). The bars represent the mean ± standard error of the mean of 5–13 rats/group.

FSL rats that were treated systemically with the H_3_ receptor antagonist clobenpropit had significantly less immobility time compared with saline-treated FSL rats (*t*_12_ = 2.634, *p* = 0.022; Figure 1A, left). To test the involvement of the hippocampus in this antidepressant effect, we administered clobenpropit locally by direct injection into the hippocampus. Clobenpropit significantly reduced immobility time compared to ACSF-injected control rats (*t*_9_ = 3.917, *p* = 0.003; Figure 1A, right), suggesting that blocking H_3_ receptors selectively in the hippocampus is sufficient to yield an antidepressant-like response in FSL rats.

### Clobenpropit Reverses Impaired Memory in FSL Rats

In the passive avoidance test, treating FSL rats with clobenpropit increased the latency time such that none of the FSL rats crossed to the dark-shock compartment (Fisher’s exact test *p* = 0.036; Figure 1B). In the novel object recognition test, baseline locomotor activity was lower in FSL compared to Sprague-Dawley rats (distance: *t*_11_ = 2.79, *p* = 0.017), but was not affected by clobenpropit treatment (distance: *t*_11_ = 1.19, *p* = 0.26; velocity: *t*_11_ = 0.87, *p* = 0.4). Clobenpropit treatment did not affect the exploration time during the training phase (Figure 1C, session 1). During the test session, saline-treated Sprague-Dawley rats preferentially explored the novel object (*t*_8_ = 3.82, *p* = 0.005), whereas the saline-treated FSL rats showed no clear preference (Figure 1C). Treating FSL rats with clobenpropit increased the recognition index to Sprague-Dawley levels in comparison to saline-treated FSL rats (*t*_8_ = 2.28, *p* = 0.052), indicating that treatment restored recognition memory in FSL rats (Figure 1C). Together, these results confirm that blocking H_3_ receptors improved memory in a rat model of depression with memory deficits.

### The Anxiety-Related Phenotype in FSL Rats is Not Affected by Clobenpropit

**Figure 2.**
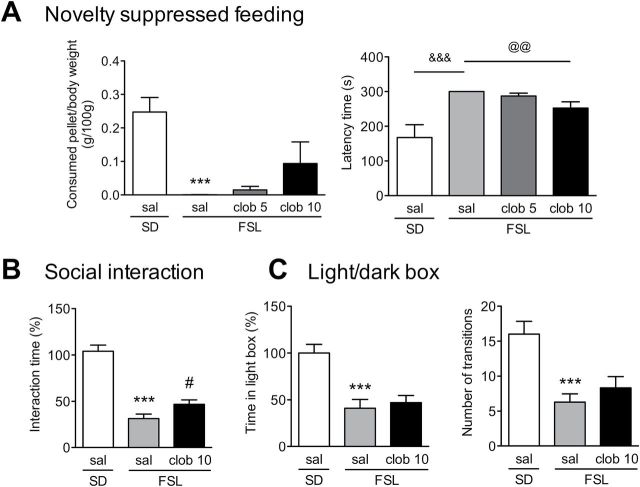
Clobenpropit does not affect anxiety-like behavior in Flinders Sensitive Line (FSL) rats. (A) Novelty suppressed feeding test. The left panel shows the amount of a food pellet (normalized to the body weight) that was consumed during 5min. The right panel shows the latency (in seconds) until the rat began to eat the pellet. Note that none of the saline-treated FSL rats began to eat the pellet within the 300-s observation period, explaining the lack of an error bar in this group. Where indicated, the rats were injected subcutaneously with either saline (sal) or clobenpropit (clob; 5 or 10mg/kg). (B) Social interaction test. The bars represent the total interaction time between pairs of rats during a 5-min trial; the data were normalized to the saline-injected Sprague-Dawley (SD) rats (100%). Rats were injected with either saline or 10mg/kg clobenpropit. (C) Light/dark box test. The left panel shows the total time spent in the light compartment during the 5-min test, normalized to the saline-injected Sprague-Dawley rats (100%). The right panel shows the total number of transitions between the dark and light compartments during the 5-min test. Rats were injected with either saline or 10mg/kg clobenpropit. In all three tests (A–C), the FSL rats had increased anxiety-related behavior compared to Sprague-Dawley rats, and this was not reversed by clobenpropit. ***p < 0.001 vs. the saline-injected Sprague-Dawley group (Student’s *t*-test); ^#^ *p* < 0.05 vs. the saline-injected FSL group (Student’s *t*-test); ^&&&^ *p* < 0.001 (Fisher’s exact test); and ^@@^ *p* < 0.01 (Chi-square test for trend on all three FSL groups). The bars represent the mean ± standard error of the mean of 7 rats/group.

Previous studies testing the FSL rats in anxiety-related paradigms have reported conflicting results in anxiety-like behaviors, with no or reduced anxiety and reduced social interaction (Overstreet et al., 1995, 2004; Abildgaard et al., 2011). We measured the basal anxiety levels of FSL rats—and the effect of clobenpropit treatment—using three anxiety-related tests: the novelty suppressed feeding test, the social interaction test, and the light/dark box test. In the novelty suppressed feeding test, FSL rats consumed less food compared to Sprague-Dawley rats (*t*_12_ = 6.34, *p* < 0.001; Figure 2A, left) and had a longer delay before eating (Fisher’s exact test *p* < 0.001; Figure 2A, right). Clobenpropit treatment did not significantly affect food consumption (Figure 2A) and was associated with a small decrease of the latency time (Chi-square = 6.98, df = 1, *p* = 0.008). In the social interaction test, the saline-treated FSL rat pairs spent significantly less time interacting compared to saline-treated Sprague-Dawley rats (*t*_12_ = 8.79, *p* < 0.001), and clobenpropit slightly increased FSL rat interaction time (*t*_11_ = 2.24, *p* = 0.045; Figure 2B). In the light/dark box test, the FSL rats spent significantly more time in the dark compartment (*t*_12_ = 4.46, *p* < 0.001) and had fewer light-dark transitions (*t*_12_ = 4.46, *p* < 0.001) compared to the Sprague-Dawley rats, and this anxiety-related behavior was not affected by clobenpropit treatment (Figure 2C). Taken together, these results show that FSL rats have a robust anxiety-related phenotype that is not reversed by clobenpropit treatment.

### GluN2A Levels are Increased by Clobenpropit Treatment

**Figure 3.**
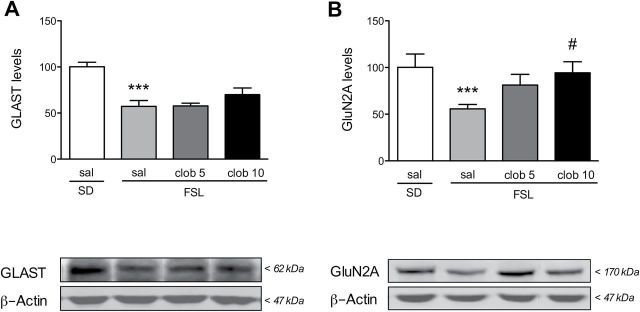
Clobenpropit increases hippocampal GluN2A—but not GLAST—protein levels. Rats were treated with either saline (sal) or the indicated dosage of clobenpropit (clob), and (A) hippocampal GLAST and (B) the NMDA receptor GluN2A subunit were measured using Western blot analyses. Protein levels were normalized to ß-actin, and each normalized level was then normalized to the saline-injected Sprague-Dawley (SD) group (100%). The levels of both GLAST and GluN2A were lower in the Flinders Sensitive Line (FSL) rats compared to Sprague-Dawley rats, but only GluN2A levels were increased to Sprague-Dawley levels following clobenpropit treatment. ***p < 0.001 vs. the saline-injected Sprague-Dawley group (Student’s *t*-test); ^#^ *p* < 0.05 vs. the saline-injected FSL group (Dunnett’s multiple comparison post hoc test). The bars in A and B represent the mean ± standard error of the mean of 7 rats/group. The lower panels show representative immunoblots.

We recently reported that FSL rats have reduced expression of the astrocytic glutamate transporter GLAST and increased glutamatergic transmission (Gómez-Galán et al., 2013). Here, we found that clobenpropit treatment did not significantly increase the expression of GLAST in the hippocampus of FSL rats (Figure 3A, left). The other astrocytic glutamate transporter, GLT1, is not changed in the FSL compared to Sprague-Dawley rats and we did not observe any compensatory increase with clobenpropit (Figure S2A).

The level of the N-methyl-D-aspartate (NMDA) receptor subunit GluN2A was significantly reduced in FSL rats compared to Sprague-Dawley (*t*_11_ = 3.18, *p* = 0.009), and clobenpropit treatment increased the amount of GluN2A (ANOVA *F*_2,17_ = 4.27, *p* = 0.031; Figure 3B). No change was detected in the glutamate receptor subunits GluA1, GluA2, GluN1, or GluN2B (Figure S2B–E) in either group.

### Clobenpropit Does Not Modulate CA1 Glutamatergic Input

**Figure 4.**
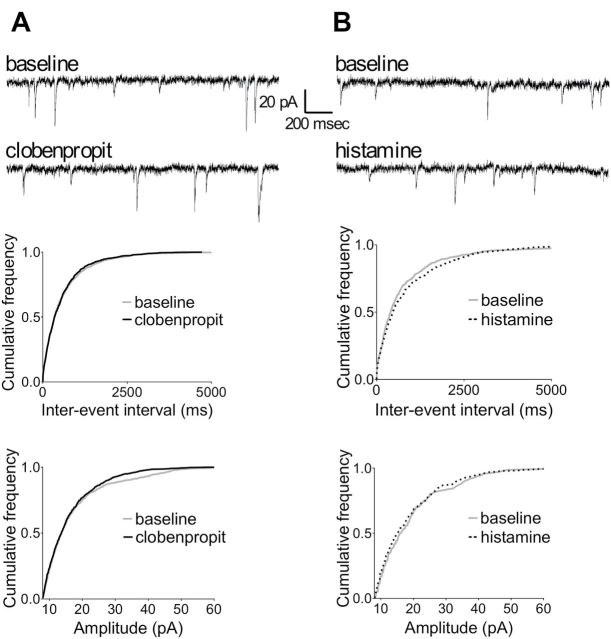
Neither clobenpropit nor histamine affects excitatory postsynaptic currents. The upper panels show representative traces recorded before (baseline) and during the application of (A) clobenpropit or (B) histamine. The lower panels show the cumulative frequency distributions of the inter-event interval (IEI) in ms and amplitude in pA of the excitatory postsynaptic currents. Neither clobenpropit nor histamine affected the IEI or amplitude as determined using the Kolmogorov-Smirnov test. Clobenpropit IEI: *p* = 0.91; clobenpropit amplitude: *p* = 0.13 (n = 5 recordings); histamine IEI: *p* = 0.26; histamine amplitude: *p* = 0.57 (n = 4 recordings).

Next, we performed whole-cell patch clamp recordings from CA1 pyramidal neurons in hippocampal slices in order to test whether clobenpropit could reverse the increased glutamatergic transmission that we observed previously in FSL rats (Gomez-Galan et al., 2013). Applying clobenpropit to the bath solution did not affect either the frequency or amplitude of spontaneous EPSCs (Figure 4A). Because H_3_ receptors are generally presynaptic and provide negative control of neurotransmitter release, clobenpropit might exert its effects by disinhibiting the release of histamine. Therefore, to exclude the possibility that spontaneous histamine release is not sufficient for clobenpropit to exert an observable effect, we tested whether histamine itself can modulate EPSCs; however, applying histamine to the slices did not affect either the frequency or amplitude of the EPSCs (Figure 4B).

### Clobenpropit Enhances LTP in FSL Rats Through Histamine H_1_/H_2_ Receptor Activation

**Figure 5.**
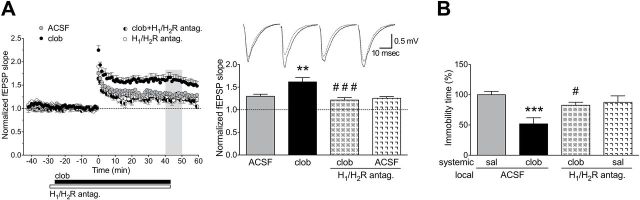
Clobenpropit (clob) increases synaptic plasticity and decreases the depressive phenotype in Flinders Sensitive Line (FSL) rats through H_1_/H_2_ histamine receptor activation. (A) Synaptic plasticity in hippocampal slices from FSL rats. The left panel shows the average normalized field excitatory postsynaptic potentials (fEPSPs) slope over time. High-frequency stimulation (HFS) was applied at time 0, and the application of the drugs to the bath is indicated by the horizontal bars. The right panel shows the average normalized fEPSP slopes 40–50min following HFS (indicated by the shaded box in the left panel). Insert represents average fEPSP from one representative slice recorded at baseline (dashed line) and following HFS (solid line). Clobenpropit (n = 6 slices) enhanced long-term synaptic potentiation (LTP) compared to control (artificial cerebrospinal fluid [ACSF]-treated) slices (n = 6 slices), and this effect was blocked by the addition of the H_1_ and H_2_ receptor antagonists trans-triprolidine and cimetidine, respectively (clob + H_1_/H_2_R antag., n = 5 slices). Application of H_1_/H_2_ receptor antagonists alone did not affect LTP (ACSF + H_1_/H_2_R antag., n = 6). The data are presented as mean ± standard error of the mean; **p < 0.01 vs. ACSF; ^###^ *p* < 0.001 vs. clobenpropit (Bonferroni’s post hoc test). (B) Forced swim test. Total immobility time was normalized to the group that received local (hippocampal) ASCF and systemic (subcutaneous) saline injections (100%). Local hippocampal injections of the H_1_/H_2_ receptor antagonists blocked the antidepressant effect of clobenpropit. ***p < 0.01 vs. saline/ACSF; ^#^ *p* < 0.05 vs. clobenpropit/ASCF (Bonferroni’s post hoc test). The bars represent the mean ± standard error of the mean of 5–7 rats/group. Saline, sal.

We tested whether clobenpropit affects CA3-CA1 synaptic plasticity in acute hippocampal slices. Bath application of clobenpropit to FSL hippocampal slices increased LTP compared to control FSL slices (Figure 5A). To determine whether this effect was dependent on a disinhibition of histamine release and the subsequent activation of H_1_ and H_2_ histamine receptors, we repeated these LTP experiments in the presence of H_1_ and H_2_ receptor antagonists (trans-triprolidine and cimetidine, respectively). Although bath application of trans-triprolidine and cimetidine alone did not reduce LTP, this treatment completely prevented the increased LTP induced by clobenpropit (Figure 5A). Indeed, the two-way ANOVA showed an effect of clobenpropit application (F_1,19_ = 4.83, *p* = 0.04), of the H_1_/H_2_R block (F_1,19_ = 12.64, *p* = 0.002), and of the interaction of clobenpropit x H_1_/H_2_R block (F_1,19_ = 8.06, *p* = 0.01). Clobenpropit’s effect was not mediated by a general increase in fEPSPs, as clobenpropit did not alter the baseline (i.e. pre-LTP induction) fEPSP slope compared to control (ACSF) treatment (Figure S3A). To test whether the ability of clobenpropit to increase LTP is selective for rats with intrinsically impaired plasticity, we measured the effect of clobenpropit on LTP induced in hippocampal slices from Sprague-Dawley rats (Figure S3B). Clobenpropit did not increase LTP in Sprague-Dawley rats.

### Histamine Plays a Role in the Antidepressant Effect of Clobenpropit

Our electrophysiology experiments suggest that clobenpropit enhances synaptic plasticity by increasing the release of histamine. To test whether the same mechanism underlies the antidepressant effect of clobenpropit, FSL rats were treated systemically with clobenpropit (or saline) together with hippocampal injections of H_1_ and H_2_ receptor antagonists (or ACSF). Following treatment, the rats were subjected to the forced swim test. Hippocampal injection of trans-triprolidine and cimetidine blocked the antidepressant-like effect of systemic clobenpropit (Figure 5B). Indeed, the two-way ANOVA showed an effect of clobenpropit systemic treatment (F_1,19_ = 9.45, *p* = 0.006) and an interaction of clobenpropit x local H_1_/H_2_R block (F_1,19_ = 6.27, *p* = 0.022). These results suggest that clobenpropit’s antidepressant effect is mediated by disinhibition of histamine release and subsequent activation of postsynaptic H_1_ and H_2_ receptors specifically in the hippocampus. To test whether the H_1_ and the H_2_ receptors contributed differently to the antidepressant effect of clobenpropit, either trans-triprolidine or cimetidine (or ACSF) were locally injected in the hippocampus in clobenpropit-treated FSL rats. The block of either H_1_ or H_2_ receptors did not increase the immobility time in clobenpropit-treated FSL rats compared to local ACSF-injected rats (Figure S3C). This suggests that the activation through endogenous histamine of either receptor by itself is still sufficient to mediate the antidepressant effect of clobenpropit.

## Discussion

FSL rats, a model of depression, exhibit behavioral despair in the forced swim test, as well as memory deficits (Eriksson et al., 2011; Gómez-Galán et al., 2013; Overstreet and Wegener, 2013). Here, we report that the H_3_ receptor antagonist clobenpropit treats both of these behaviors effectively and, most importantly, the hippocampus is harboring the antidepressant mechanism. The fact that clobenpropit did not affect baseline locomotor activity indicates that clobenpropit’s effect is not due to unspecific changes of locomotor activity. The memory-enhancing effect of clobenpropit is in line with the well-known pro-cognitive effects of H_3_R antagonists (Huang et al., 2004; Yu et al., 2006; Esbenshade et al., 2008). Previously-described anxiety tests in FSL rats have not given a consistent result (Overstreet et al., 1995, 2004; Abildgaard et al., 2011); thus, we tested the FSL rats in anxiety-related paradigms and examined the effect of clobenpropit on anxiety. Our results show that FSL rats have increased anxiety-like behavior compared to Sprague-Dawley rats, the control strain from which the FSL line was originally derived (Overstreet et al., 1986). A reduction in baseline locomotor activity and object-targeted exploration has already been described in FSL rats (Overstreet, 1986; Gómez-Galán et al., 2013), and may represent a potential bias in behavioral tests; thus, we included anxiety-related measures that are unlikely to be affected by differences in locomotor activity (pellet consumption, number of animals eating during the test observation, dark/light compartment preference). The FSL rat’s anxiety-like behavior was only slightly affected by clobenpropit, which is consistent with the finding that neither H_3_R agonists nor H_3_R antagonists affect anxiety in Sprague-Dawley rats (Pérez-García et al., 1999). On the other hand, in specific experimental conditions, inhibiting H_3_ receptors can reduce or increase anxiety; for example, Mohsen et al. (2014) showed that the H_3_R antagonist JNJ-10181457 was anxiogenic in mice. However, H_3_R-knockout mice have reduced anxiety-like behavior (Rizk et al., 2004), and injecting an H_3_R antagonist directly into the lateral septum decreases anxiety-like behavior in wild-type mice (Chee and Menard, 2013). A recent study shows clear anxiolytic activity in mice using a imidazole-free H_3_R antagonist (Bahi et al., 2014). It is suitable that the levels of histamine available in the brain determine a differential response in modulating the anxiety levels. In addition, anxiety is also modulated by other neurotransmitter systems that can be tuned by H_3_R antagonism as part of hetero-receptor function. For example, increasing glutamate levels induces anxiety-related behavior (Harvey and Shahid, 2012; Marrocco et al., 2012; Rey et al., 2012; Grivas et al., 2013). Therefore, the dose, affinity, and potency of the compound tested and the genetic background of the animal may determine these behavioral differences.

Interestingly, in our study injecting clobenpropit directly into the CA1 area of the hippocampus was sufficient to produce a significant antidepressant effect in FSL rats, and the role of the hippocampus in the effect of clobenpropit was confirmed further by the finding that blocking both H_1_ and H_2_ receptors selectively in the same hippocampal area prevented the antidepressant effect of systemic clobenpropit. The CA1 area of the hippocampus contains a high density of histamine H_1-3_ receptors (Vizuete et al., 1997; Pillot et al., 2002), but low levels of the H_4_ receptor (Connelly et al., 2009). Our results are in good agreement with the emerging view that the hippocampus is a key region for emotional regulation (Small et al., 2011) and plays an important role in mediating the therapeutic effect of antidepressants (Femenía et al., 2012).

 Impaired synaptic plasticity has been hypothesized to correlate with both cognitive deficits and the emotional symptoms of depression (Castren, 2013), and impaired hippocampal LTP has been shown in several models of depression (Holderbach et al., 2007; Gómez-Galán et al., 2013). In addition, the FSL rats display an increased spontaneous glutamate transmission due to reduced levels of the astrocytic glutamate transporter GLAST (Gómez-Galán et al., 2013). To further investigate clobenpropit’s mechanism of action, we measured the effect on the glutamatergic network in the hippocampus of FSL rats. Although clobenpropit enhanced hippocampal synaptic plasticity (i.e. LTP), it did not reverse the increased EPSC frequency seen in CA1 pyramidal neurons in FSL slices. Consistent with its lack of effect on EPSCs, clobenpropit did not affect the expression of GLAST in the hippocampus. These results may explain the lack of effectiveness in decreasing the anxiety levels in FSL rats (Pittenger et al., 2008; Krystal et al., 2010).

On the other hand, clobenpropit restored the reduced expression of the NMDA receptor subunit GluN2A in the hippocampus of FSL rats. Our current finding of reduced hippocampal GluN2A levels in FSL rats is consistent with previous reports (Eriksson et al., 2011), but not with our own previous observations (Gómez-Galán et al., 2013). This discrepancy might be explained by the highly dynamic nature of GluN2A expression (Yashiro and Philpot, 2008). It is likely that FSL rats, which are more vulnerable to stress (Pucilowski et al., 1993), are particularly sensitive to variations of this subunit. Regardless of the absolute GluN2A levels in the FSL rats versus Sprague-Dawley rats, the clobenpropit-induced increase in hippocampal GluN2A levels is consistent with the clobenpropit-induced enhancement of memory and hippocampal LTP. It is unlikely that an increase in GluN2A levels underlies the increase in LTP; rather, GluN2A translation might be induced in parallel with AMPA receptor insertion (the classic mechanism of LTP; Udagawa et al., 2012) and entail an increase in both signal amplitude and fidelity (Singh et al., 2011).

Histamine can bind directly to NMDA receptors, thereby increasing plasticity (Brown et al., 1995). However, we found that both clobenpropit-induced LTP enhancement and clobenpropit’s antidepressant effect were prevented by treatment with H_1_ and H_2_ receptor antagonists (Figure 5). These results illustrate that histamine receptors have a direct role in increasing plasticity in FSL rats, which is consistent with studies that demonstrated antidepressant and plasticity-enhancing effects of histamine via H_1_ and H_2_ receptors (Lamberti et al., 1998; Giovannini et al., 2003; Zlomuzica et al., 2009; Luo and Leung, 2010). Moreover, these results, together with the fact that clobenpropit did not affect glutamate synaptic activity (Figure 4), suggest that clobenpropit does not exert its antidepressant effect by reducing glutamate transmission, but rather through disinhibiting histamine release.

In addition to being an H_3_R antagonist, clobenpropit can act as a H_3_R inverse agonist and a H_4_R agonist. Here we show that the antidepressant effect of clobenpropit is blocked by the H_1_ and H_2_ receptor antagonists, suggesting that clobenpropit acts through reduced activity of the presynaptic histamine H_3_ receptors and increased histamine release. This indicates that post-synaptic H_3_ receptors or H_4_ receptors are not involved. However, in our experimental setting, with the presence of endogenous histamine, we cannot discriminate between the effects of an inverse agonist or antagonist.

Taken together, this work clearly demonstrates that H_3_ receptors selectively affect mechanisms that underlie cognitive and depressive-like behavior in a model of depression. In combination with clobenpropit’s plasticity-enhancing effect, these results underscore the important role of increasing plasticity in mediating the effects of antidepressants (Vetencourt et al., 2008; Castren, 2013), especially in the hippocampus. However, our study focused on the acute effects of clobenpropit, and does not provide information regarding the persistence of plasticity changes necessary for an effective antidepressant effect. Nevertheless, the ability of clobenpropit to target specific biological mechanisms—and thereby selectively treat specific behavioral deficits—increases our understanding of H_3_ receptor antagonists as therapeutic targets for several symptoms of depressive disorders. Moreover, our results may serve as a starting point for developing biomarkers to identify specific symptoms based on mechanistic knowledge.

## Supplementary Material

For supplementary material accompanying this paper, visit http://www.ijnp.oxfordjournals.org/

## Statement of Interest

The authors declare no conflicts of interest.

## Supplementary Material

Figure S1A
