# Supplementary figures and images for: Hippocampal-Dependent Antidepressant Action of the H3 Receptor Antagonist Clobenpropit in a Rat Model of Depression
Source: Int J Neuropsychopharmacol. 2015 Apr 27;18(9):pyv032. doi: 10.1093/ijnp/pyv032 (PMC4576519; doi:10.1093/ijnp/pyv032)

# Supplementary Figure 3

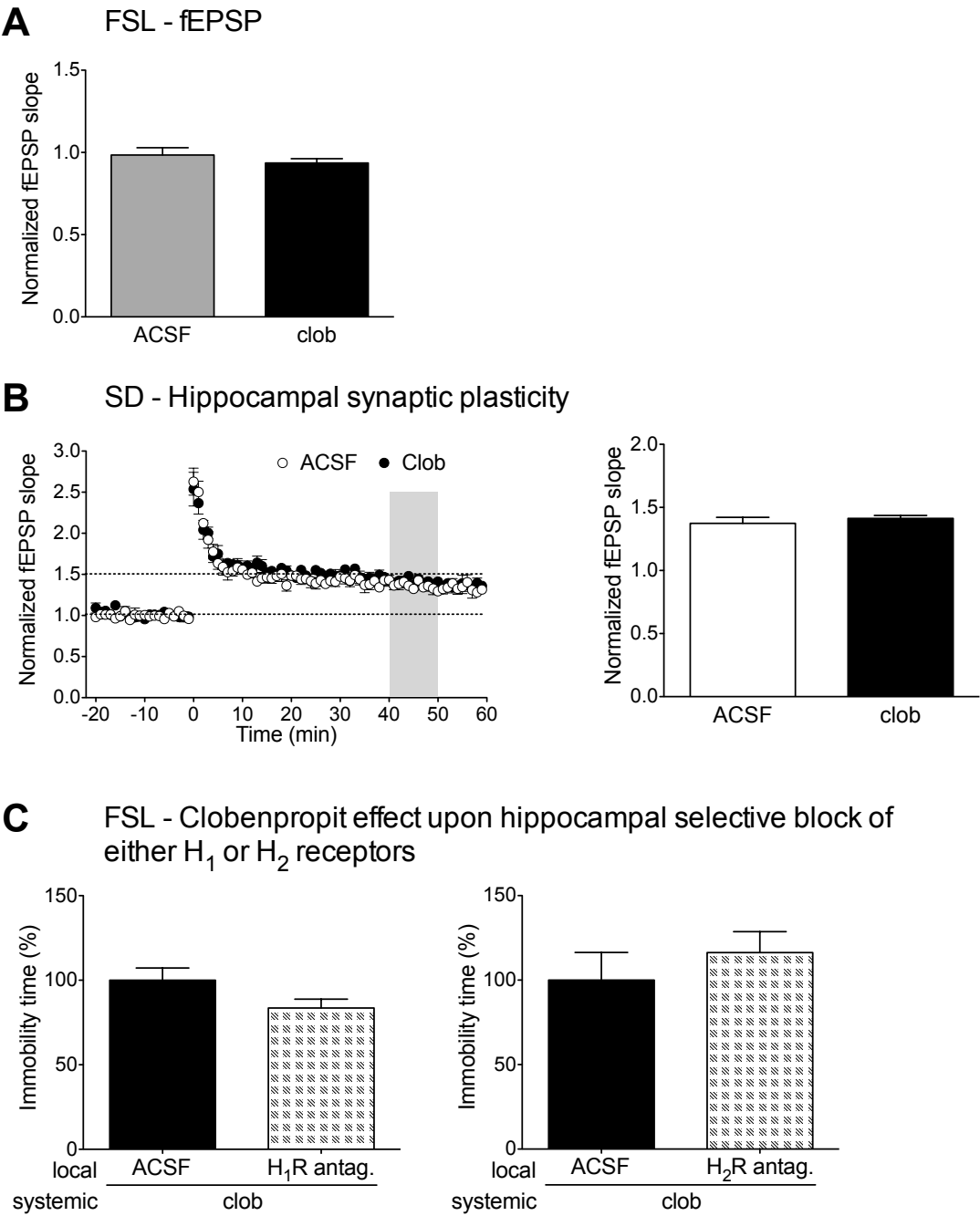

Supplement: Figure S1A [file Suppl_figure_3.pdf]

# Supplementary Figure 2

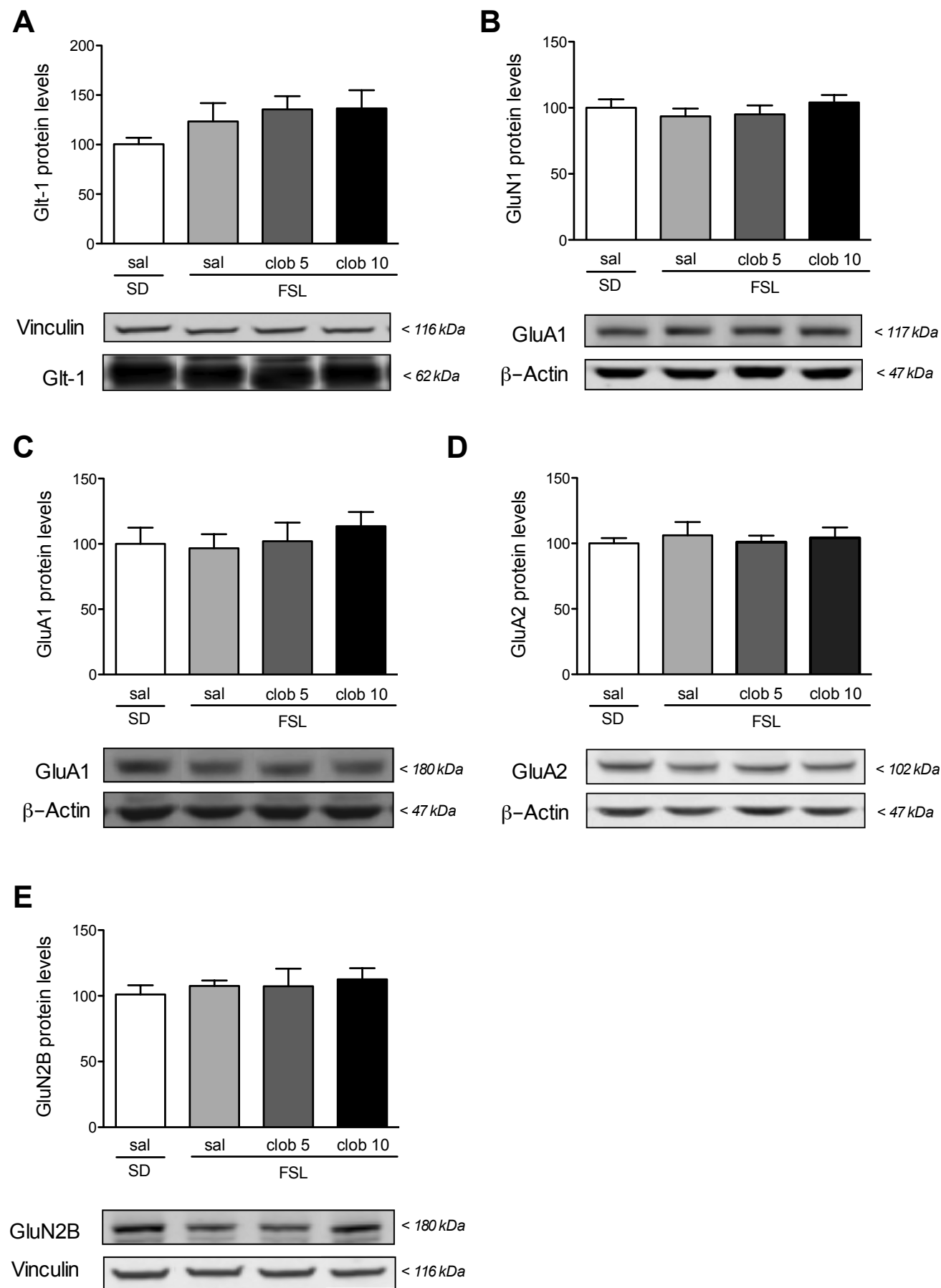

Supplement: Figure S1A [file Suppl_figure_2.pdf]
