# Supplementary material for: Hippocampal-Dependent Antidepressant Action of the H3 Receptor Antagonist Clobenpropit in a Rat Model of Depression
Source: Int J Neuropsychopharmacol. 2015 Apr 27;18(9):pyv032. doi: 10.1093/ijnp/pyv032 (PMC4576519; doi:10.1093/ijnp/pyv032)
Supplement: Figure S1A [file pyv032_Supplementary_Figure_legends.docx]

Supplementary Figure 1

A. Schematic overview of the novel object recognition test. S0 represents the 20-min habituation stage one day prior to performing the test. In session S1, “A” and “A1” represent the two identical objects; in session S2, “A” and “B” represent the familiar and novel objects, respectively (also referred to as “F” and “N”, respectively, in the main text). B. Schematic overview of the passive avoidance test. In the training session, “US” indicates the unconditioned stimulus (a 2-sec, 0.4-mA shock delivered through the floor grid). C. Cannulae placement for directly injecting drugs to the hippocampus. Upper-left panel: drawing of a coronal brain section, showing the guide cannulae implanted bilaterally above the CA1 fields in the posterior dorsal hippocampus (mediolateral: ±3.0 mm; anteroposterior: −4.2 mm; dorsoventral: −1.3 mm; coordinates are relative to bregma and the dura surface, with a 0° angle from the vertical axis in the coronal plane). The coordinates are based on the Rat Brain Atlas (Paxinos and Watson, 1998). The location of the implantation site was histologically confirmed post-mortem (upper-right and lower panels). The images show a representative cannula placement at increasing magnification (1x, 2x, and 4x scale bar represents 1 mm). The circle in the 2x magnification picture describes the area where the cannulae have been implanted.

Supplementary Figure 2

Glutamate receptor protein levels in the hippocampus following systemic clobenpropit treatment. Each panel summarizes the protein levels of the indicated receptor subunit normalized to ß-actin or vinculin, then normalized to saline-injected Sprague-Dawley rats (100%). Sprague-Dawley rats were injected with saline, and FSL rats were injected with saline, 5 mg/kg or 10 mg/kg clobenpropit. A–E. Bars represent the densitometry analysis for protein levels of GLT-1 glutamate astrocytic transporter, the AMPA receptor subunits GluA1 and GluA2, the NMDA receptor subunit NR1 and NR2B, expressed as percentage of change respect to the Sprague-Dawley rats. Values represent the mean±SEM of 7 rats/group. No significant differences were observed in any of these proteins. Below each panel are representative immunoblots for proteins and their corresponding loading control (ß-actin or vinculin) from hippocampal homogenates of SD saline, FSL saline, FSL clobenpropit at 5 and 10 mg/kg.

Supplementary Figure 3

A. Clobenpropit does not affect baseline hippocampal fEPSPs in FSL rats. fEPSP slopes are normalized to the baseline average slope for each slice (-30 min before HFS; see Figure 5). The panel shows the mean±SEM of averaged, normalized fEPSP slopes in the 10 min prior to HFS for clobenpropit- and control ACSF-treated slices. Clobenpropit did not affect fEPSP slope. B. Clobenpropit does not affect hippocampal synaptic plasticity in Sprague-Dawley rats. The left panel shows average (mean±SEM) normalized fEPSP slopes. High-frequency stimulation (HFS) was applied at time 0. The right panel shows the mean±SEM of averaged, normalized fEPSP slopes recorded 40–50 min following HFS (indicated by the shaded box in the left panel). C. Neither the H_1_ nor the H_2_ receptor antagonist increases the immobility time in clobenpropit-treated FSL rats. Total immobility time was normalized to the respective control group that received local (hippocampal) ASCF injections (100%). All groups received systemic (subcutaneous) clobenpropit administrations. The bars represent the mean±SEM of 6–8 rats/group.
